# Out-of-pocket payment for surgery in Uganda: The rate of impoverishing and catastrophic expenditure at a government hospital

**DOI:** 10.1371/journal.pone.0187293

**Published:** 2017-10-31

**Authors:** Geoffrey A. Anderson, Lenka Ilcisin, Peter Kayima, Lenard Abesiga, Noralis Portal Benitez, Joseph Ngonzi, Mayanja Ronald, Mark G. Shrime

**Affiliations:** 1 Department of Surgery, Massachusetts General Hospital, Boston, Massachusetts, United States of America; 2 Program in Global Surgery and Social Change, Department of Global Health and Social Medicine, Harvard Medical School, Boston, Massachusetts, United States of America; 3 Department of Surgery, Mbarara University of Science and Technology, Mbarara, Uganda; 4 Department of Obstetrics and Gynecology, Mbarara University of Science and Technology, Mbarara, Uganda; Duke University, UNITED STATES

## Abstract

**Background and objectives:**

It is Ugandan governmental policy that all surgical care delivered at government hospitals in Uganda is to be provided to patients free of charge. In practice, however, frequent stock-outs and broken equipment require patients to pay for large portions of their care out of their own pocket. The purpose of this study was to determine the financial impact on patients who undergo surgery at a government hospital in Uganda.

**Methods:**

Every surgical patient discharged from a surgical ward at a large regional referral hospital in rural southwestern Uganda over a 3-week period in April 2016 was asked to participate. Patients who agreed were surveyed to determine their baseline level of poverty and to assess the financial impact of the hospitalization. Rates of impoverishment and catastrophic expenditure were then calculated. An “impoverishing expense” is defined as one that pushes a household below published poverty thresholds. A “catastrophic expense” was incurred if the patient spent more than 10% of their average annual expenditures.

**Results:**

We interviewed 295 out of a possible 320 patients during the study period. 46% (CI 40–52%) of our patients met the World Bank’s definition of extreme poverty ($1.90/person/day). After receiving surgical care an additional 10 patients faced extreme poverty, and 5 patients were newly impoverished by the World Bank’s definition ($3.10/person/day). 31% of patients faced a catastrophic expenditure of more than 10% of their estimated total yearly expenses. 53% of the households in our study had to borrow money to pay for care, 21% had to sell possessions, and 17% lost a job as a result of the patient’s hospitalization. Only 5% of our patients received some form of charity.

**Conclusions and relevance:**

Despite the government’s policy to provide “free care,” undergoing an operation at a government hospital in Uganda can result in a severe economic burden to patients and their families. Alternative financing schemes to provide financial protection are critically needed.

## Introduction

Recently the Lancet Commission on Global Surgery (LCoGS) recommended that every country in the world measure six surgical metrics as indicators of the strength of its surgical system [1]. In 2016 four of these indicators were included in the World Bank’s list of important development metrics, the World Development Index (WDI) [2]. Indicators 5 and 6 are the rate at which patients in a given country experience a catastrophic or impoverishing expenditure as a result of having an operation [1]. Despite these metrics being included in the WDI, little information exists about them in the literature.

The LCoGS is advocating for a scale-up of surgery around the world, especially in Low and Middle-Income Countries (LMIC’s). The Lancet Commission notes that, based on global burden of disease estimates, an additional 143 million operations are needed globally each year [3]. Furthermore, only six percent of the operations performed every year happen in the poorest regions of the world [4]. Much work has been done to dispel the myth that surgical care is not “cost effective” in LMIC’s [5]. While surgical interventions are considered “best buys” for healthcare systems, receiving an operation can be economically devastating for the individual. If there is a massive, global increase in surgical volume without a concurrent improvement in financial protection, the end result will be a considerable increase in poverty and economic catastrophe. This is even more tragic because this financial devastation will disproportionately affect the poorest among us [6].

In order to predict the economic consequences of a scale-up of surgical services, information is needed about the current economic impacts of surgical care. This type of information would also help Ministries of Health and Finance make policy decisions about healthcare funding, financial protection strategies and resource allocation. One study from eastern Uganda calculated the overall costs of several common procedures and determined them to be similar to the treatment of Human Immunodeficiency Virus/ Acquired Immunodeficiency Syndrome (HIV/AIDS) or Tuberculosis (TB). However, it did not examine costs to patients [7]. According to the World Bank, about 40% of total Ugandan health care expenditures are out-of-pocket (OOP), and modeled data suggests nearly 80% of the population are at risk of catastrophic or impoverishing expenditures due to surgical care [2, 6].

All care delivered at government hospitals in Uganda is completely free of charge. There are no doctor’s fees or hospital bills. However, if there are out-of-stock items or if equipment is broken or otherwise unavailable, then patients and their families must purchase those goods and services outside of the government facility before care can be rendered. For instance, if the hospital is out of gloves, bandages and antibiotics, then a family member must go to the local pharmacy to purchase these items and bring them back to the hospital so they can be used to treat the patient. Stock-outs happen with great frequency at many government facilities around Uganda and in other developing countries [8, 9].

The goal of this study was to measure the economic burden associated with seeking surgical care at a government hospital in Uganda. To achieve this end, we interviewed patients who were being discharged from the hospital after receiving an operation and asked them about their out-of-pocket expenses for surgery.

## Methods

### Settings

This study was conducted at Mbarara Regional Referral Hospital (MRRH), a 600 bed government hospital that serves as the only referral center for southwest Uganda, a region of 8 million people [10, 11]. The hospital has 4 operating rooms, 12 obstetrician/gynecologists, 11 surgeons, and 6 anesthesiologists. It performs 6,000–8,000 operations annually. MRRH is associated with Mbarara University of Science and Technology (MUST) which has a medical school, nursing school and a variety of residency training programs. The hospital is situated in the town of Mbarara, about 4 hours southwest of the capital city of Kampala and is surrounded by a large rural area.

### Study population

Over 3 weeks in April 2016 we interviewed patients and/or their attendants as they were being discharged from MRRH after having an operation.

Inclusion Criteria: Any patient on any service that had a procedure in the operating theatre during this admission was eligible for enrollment.

Exclusion Criteria: If a patient was admitted to the medical or pediatrics services post-operatively they were excluded. This accounted for only 3 patients. These patients typically had a large number of other complex medical issues that made their care less applicable to the goals of this study.

Two of the primary investigators attended daily rounds on all of the surgical services, including obstetrics and gynecology, to identify patients that were being discharged that day. If any patient was discharged outside of regular rounding time, the intern or resident responsible for the discharge notified us. Once a patient was identified, Ugandan research assistants approached them. If the patients agreed to participate, they were asked to provide written consent (or thumb print) and then were interviewed in their local language in a private office adjacent to the ward. In the case of pediatric patients, the parents provided consent and were interviewed. Each interview took approximately 15–45 minutes. Participants were compensated for participation with a bar of laundry soap valued at approximately $1.

### Determination of “poverty”

In LMIC’s, poverty is often determined by measuring household expenditure and not income. The high number of subsistence farmers in these countries makes the concept of “income” functionally irrelevant. Thus, the primary definitions of poverty by the World Bank are based on daily expenditure rather than income levels. Currently, two metrics are measured as international standards for what is considered poor. The World Bank defines “extreme poverty” as a household that spends less than $1.90/person/day and “poverty” as one that spends less than $3.10/person/day [2, 12, 13]. Both of these metrics are based on 2011 purchasing power parity (PPP), which converts various currencies to US dollar equivalents. We converted all Ugandan shillings into 2011 PPP USD by using the most recent 2011 PPP conversion factor for private consumption (1185.8 for the year 2015) from the World Bank [2].

The World Bank has data about national poverty levels which were determined from national questionnaires. We used confidence intervals to compare the baseline poverty levels among our patients with the most recent levels estimated by the World Bank (from 2012) to determine if our patient population was different from that of Uganda as a whole [2].

## Questionnaire and validation

A questionnaire was developed for this study to determine the rates of impoverishing and catastrophic expenditure after surgery. This questionnaire was compiled by drawing on information from a variety of sources (see S1 File).

The first part of the questionnaire asked participants a series of questions about their household expenditures in a typical month. The second part of the questionnaire included a series of questions about the amount of money the household had to pay, out of pocket, for this hospitalization. These questions were drawn from a literature review on the topic of catastrophic and impoverishing healthcare expenditures and combined questions from a number of well-established surveys on this topic [14–17].

We reviewed the literature on out-of-pocket payments for medical and surgical care in low resource settings and contacted authors of a number of studies to get access to previously validated survey tools [17–20]. We developed a bank of questions that could be used in our survey. We then used a modified Delphi system to validate these questions by giving sending them out to a group that included global surgery researchers, cost effectiveness analysis experts, Ugandan surgeons, former Ugandan patients, and Ugandan researchers experienced in survey instruments. We went through 2 rounds of question edits by this panel to come up with our final survey instrument. To ensure our answers were understood and being answered consistently we chose one common procedure (cesarean section) and evaluated the responses for a standard post-operative course (three- day length of stay) (S1 Fig).

### Definition of impoverishing and catastrophic expenditure

We used the established definition of an impoverishing expense as one that pushes a household into poverty. If the amount of money that a household spent in a typical year minus the amount of money they spent on this hospitalization was below the poverty line, then this was considered an impoverishing expense.

There are a number of definitions for catastrophic expenditure. The two most accepted require a household to spend: 1) more than 40% its non-subsistence expenditure, or 2) greater than 10% of its total annual expenditure on this hospitalization. We used both of these definitions in our calculations.

### Categories of healthcare expenditures

We used the three standard categories of healthcare expenditures in this study. A direct medical expense was one spent on medicine, bandages, imaging, medical supplies, studies, and informal payments. Direct non-medical expense included the money spent on transportation to the hospital and food at the hospital. Indirect expenses were primarily lost wages by the patient or attendants. We also queried indirect effects of hospitalization such as borrowing money, removal of a child from school, and job loss.

### Power calculation

Data from modeling produced for the Lancet Commission on Global Surgery, now accepted as data for the World Bank’s WDIs [2, 6], suggested that the rate of IE and CE by patients receiving surgery in Uganda were 85% and 79%, respectively. In order to have 80% power to find a 10% difference in IE with an alpha level of 0.05 we needed to interview 278 patients. By looking at MRRH data from 2014 it appeared that if we enrolled patients for three weeks we would have more than enough power to detect this level of difference.

### Data analysis

Rates of impoverishing and catastrophic expenditure were calculated as described above. Because 60% of our patients were discharged after having a cesarean section, we included a separate set of calculations based solely on this group. The 6 patients whose surgical procedure was not recorded were included in the non-cesarean group as surveys originating from the obstetrics ward were separate at the time of data entry. After adjusting all Ugandan shillings into 2011 PPP USD, summary statistics were calculated for all expense types. Given the large right sided tails, median and IQR were reported. Proportion confidence intervals for poverty and catastrophic expenditure were calculated using the Clopper-Pearson method and proportion tests were used to compare the pre and post impoverishment rates. Comparisons between cesarean patients and patients undergoing alternate surgeries were analyzed using the Wilcoxon rank-sum test for numerical data and Fisher’s exact test for categorical variables.

### Software

All statistical procedures were performed using STATA 14 (College Station, TX).

### Ethical approval

Ethical approval for this study was obtained from the Institutional Review Committee at Mbarara University of Science and Technology, the Ugandan National Committee for Science and Technology and from the Institutional Review Board at Harvard Medical School.

## Results

### Demographics

During the study period, 320 patients were discharged after having an operation and are summarized in Table 1. Of the initial 320 possible patients, nineteen left the hospital before being approached and six declined to finish the survey. We had complete or near complete interviews from a total of 295 patients. The majority of these (182) had a cesarean section. Of the remaining 113, six did not have data recorded on type of procedure. The most common other categories of surgery are listed in Table 2. It took patients a median of 60 minutes to reach the hospital once they started traveling and they stayed for a median of four days. The most common occupation of our patients was “subsistence farmer” and the median household size was four people. The median monthly household expenditure on all items was approximately $260. This is adjusted for purchasing power parity and this is what we used as a measure of poverty instead of income, consistent with international metrics.

**Table 1:** Basic demographics.

	All Patients	Cesarean patients	Other Surgery^a^ patients	p values^b^
**Number of patients**	295	182	113	
**Length of stay in days**	4 (3–5)	3 (3–4)	5 (3–13)	p < 0.0001
**Travel time in min**	60 (30–120)	60 (30–120)	90 (60–180)	p = 0.0001
**Top three occupations**	farmer 115 (39%)	farmer 62 (34%)	farmer 53 (47%)	
	business owner 47 (16%)	business owner 30 (17%)	business owner 17 (15%)	
	boda-boda driver 16 (5%)	boda-boda driver 11 (6%)	teacher 7 (6%)	
**Household size**	4 (3–5)	4 (3–5)	4 (3–6)	p = 0.02
**Monthly expenditure**^c^	259.32 (113.32–468.04)	259.91 (101.20–449.06)	251.73 (147.58–476.47)	p = 0.47

Basic demographic information collected during the survey. All data presented are medians and IQR except number of patients and top three occupations.

^a^. This includes patients undergoing all procedures other than cesarean section

^b^. p-values comparing patients undergoing cesarean section and other surgeries

^c^. Per household, estimated median monthly expenditure as converted to USD corrected for 2011 PPP

**Table 2:** Most common categories of surgical procedures.

Procedure Category	Number (%)
General Surgery	48 (16%)
Gynecologic Surgery	24 (8%)
Orthopedic Surgery	11 (4%)
Neurosurgery	10 (3%)
ENT surgery	6 (2%)

### Baseline poverty

**Fig 1:**
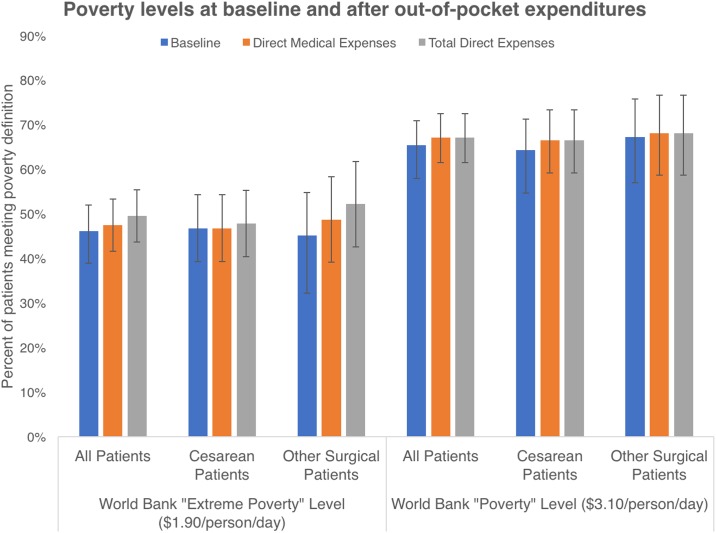
Pre and post- surgery poverty rates. Poverty Rates before and after obtaining surgical care. Poverty levels were calculated using the $1.90 and $3.10/person/day expenditures adjusted to 2011 PPP USD. While not statistically significant, this rose to 49% (p = 0.41) and 67% (p = 0.66) respectively after surgery when all costs were accounted for. Rates were slightly higher in non-surgical patients based on patient response, but again not statistically different.

The World Bank sets two international standards for poverty. The first, defined as “extreme poverty,” is spending below $1.90/person/day corrected for 2011 PPP. The second classification defines “poor” and is defined as $3.10/person/day. The most recent year for which the World Bank reports complete data for poverty is 2012. At that time an estimated 34.6% of Ugandans were impoverished based on the $1.90/person/day classification, and a full 65.0% met the $3.10/person/day poverty criteria (all patients in “extreme poverty” are also included in the above “poverty” metric) [2]. Baseline poverty statistics are summarized in Fig 1. Using these standards, 46% (95%CI 40%–52%) of our patients would be considered “extremely poor”, which is slightly higher than that estimated by the World Bank for all of Uganda. An additional 57 patients (19%) had an average expenditure of less than $3.10/person/day, for a total of 65% (95%CI 60%–71%) of patients classified as poor by the World Bank. The subset of patients who underwent cesarean sections were not significantly different at baseline from other patients (p = 0.74 for extreme poverty category, p = 0.60 for poor category (Fig 1), median monthly expenses p = 0.47 (Table 1).

### Hospital expenditures

Our patients spent a median of $75.90 (IQR 36.26–160.90) on direct medical costs alone during their hospital stay. Patients who received Cesarean section spent less, a median of $67.47 (IQR 33.73–120.59), while the other patients spent more, a median of $118.06 (IQR 42.17–320.46), p = 0.0001 (Table 3). This was primarily due to the differences in costs of medications (p = 0.0001), labs (p = 0.014) and imaging (p = 0.001). Similar expenses were incurred by both subsets due to medical supplies such as bandages, gloves and other, unspecified supplies. Approximately 10% of all patients reported making an informal payment to a hospital worker during their hospital stay. This was significantly higher for non-cesarean section patients at 15% (p = 0.024).

**Table 3:** Summary of out-of-pocket expenditures and financial impact.

	All Patients	Cesarean patients	Other Surgery Patients	P values^d^
	295	182	113	
**Median Direct Medical Expenses (IQR)**^e^	75.90	67.47	118.06	p = 0.0001
	(36.26–160.90)	(33.73–120.59)	(42.17–320.46)	
Medications^f^ (IQR)	50.60	42.17	84.33	p = 0.0001
	(25.30–126.50)	(20.24–84.33)	(37.11–168.66)	
Bandages, gloves, other supplies (IQR)	25.30	22.35	25.30	p = 0.0776
	(12.65–42.30)	(10.12–33.73)	(15.18–74.21)	
Labs (IQR)	18.97	11.38	22.77	p = 0.0144
	(8.43–35.84)	(6.75–12.65)	(8.43–42.17)	
Imaging (IQR)	25.30	25.3	50.60	p < 0.0001
	(21.08–63.25)	(21.08–25.30)	(25.30–126.50)	
Other Medical Supplies (IQR)	25.30	25.30	25.30	p = 0.4783
	(16.87–46.38)	(16.87–42.17)	(16.87–101.20)	
Made Informal payment, y (%)	28 (9.5%)	11 (6.0%)	17 (15.0%)	p = 0.024
**Median Direct Non-Medical Expenses (IQR)**	60.72	59.03	84.33	p = 0.0066
	(37.95–126.50)	(35.42–109.63)	(42.17–193.96)	
**Median Total Direct Expenses (IQR)**	155.17	137.04	248.78	p = 0.0001
	(87.70–303.59)	(87.70–211.67)	(88.54–462.98)	
Borrowed money, y (%)	157 (53%)	88 (48.4%)	69 (61.1%)	p = 0.048
Sold Possessions, y (%)	63 (21%)	26 (14.3%)	37 (32.7%)	p < 0.000
Lost job/ Unsure Job status when return, y (%)	49 (16.6%)	30 (16.5%)	19 (16.8%)	p = 0.591
Children’s education interrupted, y (%)	26 (8.8%)	13 (7.1%)	13 (11.5%)	p = 0.165
Forced to hire a professional attendant, y (%)	12 (4.1%)	2 (1.1%)	10 (8.9%)	p = 0.001
Received charity, y (%)	16 (5.4%)	8 (4.4%)	8 (7.1%)	p = 0.428

^d^. p values represent difference between patients undergoing cesarean section and other surgical patients

^e^. All monetary values are in USD corrected for 2011 PPP

^f^. Many patients could not differentiate between medications and medical supplies because they were all purchased at the same place with 1 receipt, therefore these are grouped under medications causing this number to be inflated relative to the other expenses

Direct, non-medical costs include expenses such as transport and food while hospitalized. Overall patients spent slightly less on direct non-medical costs 60.72 (IQR 37.95–126.50) than the direct medical costs. We observed similar trends as above, with cesarean patients spending less—median expenses $59.03 (IQR 35.42–109.63) and non-cesarean patients spending more $84.33 (IQR 42.17–193.96), p = 0.0066). The total median direct expenses for all patients were $155.17 (IQR 87.70–303.59). Again, patients undergoing cesarean sections paid less for their care (p = 0.0001).

Many of our patients reported adverse life events in order to pay for their care—53% had to borrow money and 21% had to sell possessions in order to pay for the expenses they incurred as a result of the hospitalization. Additionally, 16% of the households had someone lose a job and 9% had to interrupt their children’s education. Only 5% received charity to help pay for the hospital stay.

### Catastrophic expenditure

**Fig 2:**
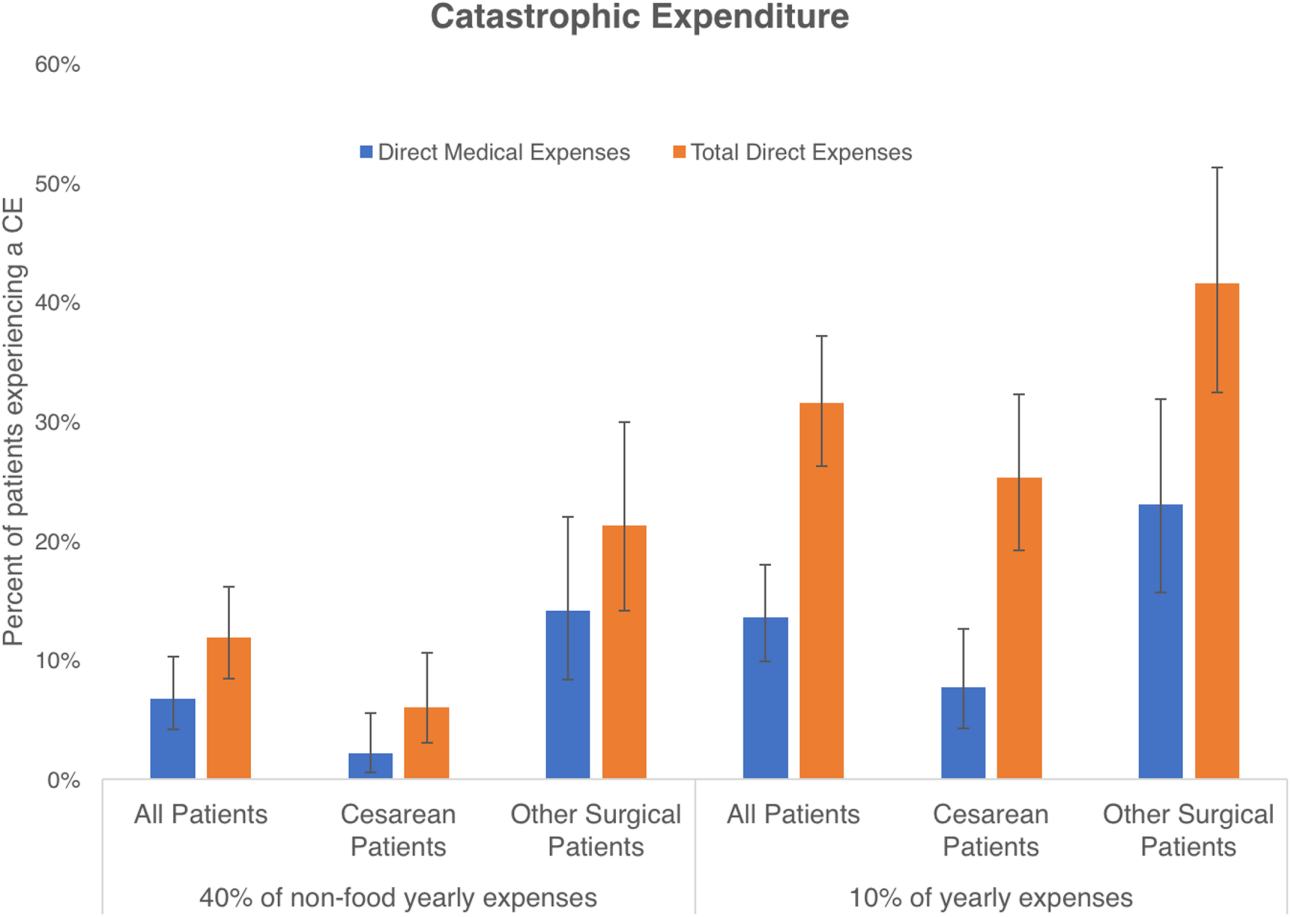
Catastrophic expenditure due to surgical care. Depending on method of calculation and whether only direct medical or all direct costs are considered, between 7% and 32% of all interviewed patients had a catastrophic expenditure. Patients who underwent a procedure other than a cesarean section were more likely to undergo a catastrophic expenditure by either definition. When calculated as in 40% of yearly non-food expenses direct medical costs were different at p = 0.0001, and total direct costs p = 0.0001; when calculated as 10% of total yearly expenses, direct medical costs were different at p = 0.0002, and total direct costs p = 0.0034.

We calculated the rate of catastrophic expenditure using the 2 most widely accepted methodologies. When defined as spending more than 40% of non-food expenses, 7% of our patients experienced an economic catastrophe from direct medical costs (Fig 2). This rose to 12% when considering all direct costs. This was higher (direct medical 14% and total direct 21%) for our non-cesarean patients compared with our cesarean patients (2% and 6%, p = 0.0009 and p = 0.0001).

When catastrophic expenditure is defined as spending greater than 10% of total annual expenses then 14% of our patients had a financial catastrophe from direct medical costs alone and 32% had one from total direct costs (Fig 2). Once again, cesarean patients were not affected as much as other patients. A catastrophic expense from total medical costs was experienced by 25% of our cesarean patients as opposed to 42% (p = 0.0034) of all the other patients.

### Impoverishing expenditure

Looking at direct medical expenses alone, 47% 95%CI (42%– 55%) (not statistically different from baseline, p = 0.74) of our patients experienced an impoverishing expense with using the World Bank’s definition of “extreme poverty”, and 67% (95%CI 61%– 72%) (p = 0.66 compared to baseline) using the $3.10/person/day “poverty” level. These levels rose to 49% (95%CI 44%–55%) (p = 0.41) and 67% (95%CI 61%–72%) (p = 0.66) respectively when all costs were accounted for (Fig 1). This represented a shift of 10 (3%) additional patients into the “extremely poor” category and 5 new patients in the poor category.

## Discussion

Accessing surgical care in Uganda is frequently economically devastating. This is true even under the best-case scenario of a government hospital with 100% “free” care. Depending on the calculation chosen, between one in eight or one in three patients who underwent surgery experienced a financial catastrophe at MRRH. This impact was even higher for patients who had an operation other than a cesarean section. Years of attention to maternal mortality by the international community has resulted in better funding for maternal health interventions, likely contributing to this disparity for non-cesarean patients. For instance, mothers who come for delivery receive a package of basic medical supplies from the government.

The patients who seek care at government facilities in Uganda have high rates of extreme poverty to begin with. In our study, 46% of patients met the World Bank’s definition of extreme poverty. While rates of new impoverishment were relatively low (5 patients were additionally considered poor, and 10 were reclassified to extreme poverty), the already high rates of poverty in this community make the high additional out-of-pocket payments unacceptable. Based on these numbers, approximately 173 patients and their families would be pushed into extreme poverty every year just for seeking surgical care at this hospital alone.

Devastating effects of paying for surgical care can also be seen in other ways [9, 16, 21]. Over half of the households in our study had to borrow money to pay for care, 21% had to sell possessions, 17% lost a job and 9% had their children’s education interrupted as a result of the hospitalization. Only 5% of our patients received some form of charity. This lack of ability to pay is all the more distressing considering more than three-quarters of the operations were surgical emergencies. The purpose of this paper was to examine the financial impact of surgery based on the new WDI definitions. The concept of poverty, however, is more nuanced and is multi-dimensional. These results begin to show some of the deeper impact that undergoing an operation can have on the lives of the patients and their families.

(Note: It is ironic that the largest source of charity was the United Nations which covered all the cost for the care received by refugees from a local camp. Their care truly was “free.”).

All care at this hospital is completely free. Patients are not charged for their hospital stay, nor do they have to pay the surgeons who treat them. Broken equipment and out-of-stock items are commonplace, however, forcing patients to pay for items at private pharmacies. During our study, the X-Ray and CT machines were broken, so all imaging had to be done at a private hospital approximately 30 minutes away. Patients frequently had to buy gloves, bandages, medicines, catheters and reagents for lab tests. All of these items were paid for directly by patients or family members prior to care being delivered. There was no special fund available for patients who were too poor to purchase these goods and services.

There are few reports in the literature regarding patients’ out-of-pocket (OOP) expenditures on surgery, and fewer still that use direct interviews rather than modeling [6, 22]. We found no other reports from Uganda that attempted to describe OOP expenditure, or rates of catastrophic or impoverishing expenses due to surgery. Most studies focusing on out-of-pocket expenditure have focused on the cost of cesarean section or used cesarean section to model remaining rates. One study in Madagascar examined data from women undergoing cesarean section to extrapolate rates of impoverishing expenditure (IE) and catastrophic expenditure (CE) for all surgery as 77.4% to 86% and 78.8% to 95.1% respectively [23]. Catastrophic expenditure due to cesarean section has been estimated at 88% for the poorest quintile in Morocco [24] and between 1.6% and 22% in a single state in India [25]. Road traffic injuries have also been a focus of OOP. While not limited to patients who underwent surgery, studies from Vietnam, Nigeria, and India report catastrophic expenditure rates after injury of 60% [20] 86% [26] and 30% [27] respectively. This study is thus one of the first to attempt to characterize impoverishing and catastrophic expenditure using primary data for all types of surgical procedures in a low- or middle-income country.

This study has several limitations that need to be kept in mind when viewing these results. This study was performed at a single institution. While the hospital in which this study was conducted is typical of government institutions in Uganda, these results are not necessarily representative of all government hospitals in Uganda specifically or other LMIC’s in general. We interviewed patients during a three-week period, which introduces bias given the variability of stock-outs of any given item. However, most of the “stock-outs” are nearly constant. For example, the CT scanner at this hospital has been broken for several years, with no known date for repair. Many expendable items were also nearly always out-of-stock.

The economic situation and healthcare financing varies widely between LMIC’s and these results are not meant to be extrapolated to all LMIC’s. Half of the medical care in Uganda is provided outside the government system at private for-profit and private not-for-profit hospitals with very different economic models from the government system. Our study interviewed patients upon discharge from the hospital. This means we were unable to capture the 2–3% of patients that die during their hospitalization, the patients that abscond prior to discharge or those patients that never even attempt to seek surgery because of the numerous barriers to care that are most intensely experienced by the poor. The inclusion of these patients would likely shift our results toward even more significant economic consequences of accessing surgery.

An additional limitation is that our survey relied on the patient’s (or their attendants) recall of their expenses. We asked patients about a “typical month’s” worth of expenditures, a standard way of obtaining these data [17, 20, 25]. In fact, in low-resource settings there are not many other ways of estimating expenditures of subsistence farmers who live in a community where receipts are rare and there is no record keeping. To further demonstrate the validity of our data we compared the baseline poverty level of our patients against Ugandan national statistics collected by the Ugandan government (Fig 1). Using the definition of poverty that is used by the Ugandan Ministry of Finance ($1/person/day expenditures), 21% of our patients were poor. The Ugandan government reports to the World Bank that 20% of its citizens are poor. Additionally, our patients spent similar amounts on individual categories of expenses (See S1 Fig).

A final limitation could be that the amount a patient reports paying could be influenced by their clinical outcome. A patient’s negative emotional associations with a poor outcome might bias them towards reporting sending more money for the hospitalization. The converse could also be true for a “good” outcome.

## Conclusions

Despite the availability of “free care,” receiving an operation at a government hospital in Uganda can result in a severe economic burden to patients and their families. The Ugandan government needs to consider alternative forms of financial protection for its citizens. If surgical care is scaled-up in Uganda, the result should not be a scale-up in financial catastrophes. The Ministry of Health and the Ministry of Finance should consider using these results, and other such studies, to help inform decisions regarding healthcare policy and resource allocation.

## Supporting information

S1 Fig Variations in reported costs for a cesarean section with standard post-operative course (3 days). These represent smoothed histogram (kernel density) of costs for the most frequent operation (cesarean section) with a standard post-operative stay (3 days). As examples of individual questions, S1a shows imaging cost, S1b shows food costs. For the total reported costs, S1c shows direct medical costs, and S1d shows total direct costs.
(TIFF)

S1 File Questionnaire. (DOCX)

S2 File Dataset. (XLSX)
