# Supplementary figures and images for: Out-of-pocket payment for surgery in Uganda: The rate of impoverishing and catastrophic expenditure at a government hospital
Source: PLoS One. 2017 Oct 31;12(10):e0187293. doi: 10.1371/journal.pone.0187293 (PMC5663485; doi:10.1371/journal.pone.0187293)

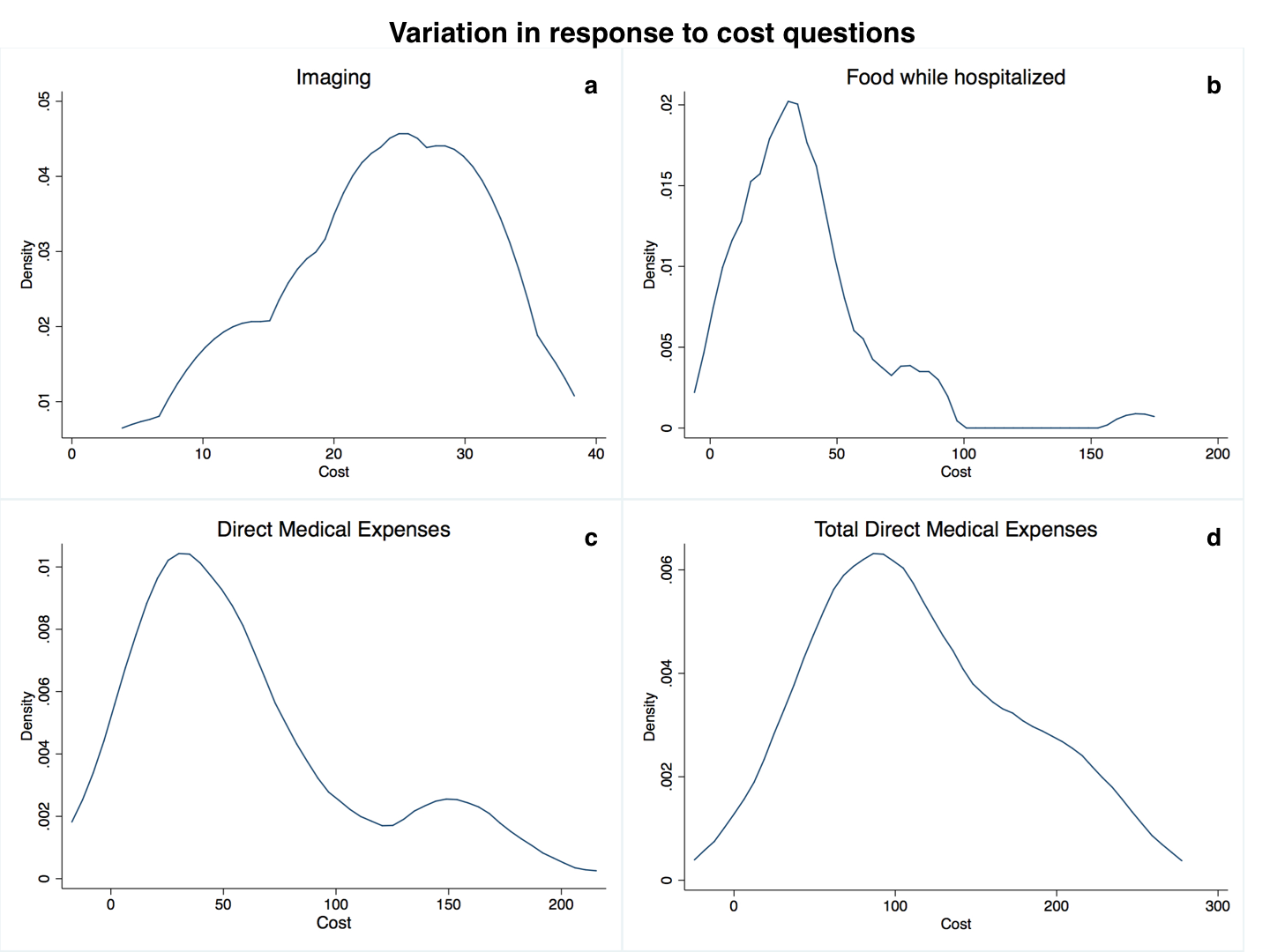

Supplement: S1 Fig — These represent smoothed histogram (kernel density) of costs for the most frequent operation (cesarean section) with a standard post-operative stay (3 days). As examples of individual questions, S1a shows imaging cost, S1b shows food costs. For the total reported costs, S1c shows direct medical costs, and S1d shows total direct costs. (TIFF) [file pone.0187293.s001.tiff]
