# Supplementary material for: Out-of-pocket payment for surgery in Uganda: The rate of impoverishing and catastrophic expenditure at a government hospital
Source: PLoS One. 2017 Oct 31;12(10):e0187293. doi: 10.1371/journal.pone.0187293 (PMC5663485; doi:10.1371/journal.pone.0187293)
Supplement: S1 File — (DOCX) [file pone.0187293.s002.docx]

**Initial Demographic Questions:**

Participant ID:

Type of Surgery:

Days in Hospital**:**

**Typical Monthly Income**

Instructions to the patient/guardian: These are a series of questions to understand the structure of your household and how much money you make per month? These questions are all based on an average month? If you cannot determine an average, tell us how much you made in the last month?

1. Where do you live (which town/ village)?
2. What is the size of your household, including yourself (how many members normally live in your house)?
3. What occupation is held by the person who makes the most money in your household *(primary breadwinner?)?*
4. How much does your household earn in an average month?

**Typical Monthly Expenditure**

Instructions to the patient/guardian: I’m going to ask you several questions about how much you spend in an average month on a variety of items. If you do not spend money on the item in question, please state zero. If you cannot determine an average, tell us how much you spent in the last month?

1. How much money does your household spend on food to eat, water, juice or soda to drink?
2. How much money does your household spend on livestock (including cows, pigs, goats, sheep, chickens etc. ) every month?
3. Excluding this hospital course, how much money does your household usually spend on health care, including medicines, fees for doctors or hospital visits, fees for traditional healers?
4. How much money does your household spend on transport?
5. How much money does your household spend on expenses for public gatherings such as weddings, parties, festivals or burials?
6. How much money does your household spend on other household matters such as clothes, improvements to your house etc?
7. How much money does your household spend on education per term for all the children you support, including school fees, books, uniforms, transport and boarding fees.
8. Do you have other monthly expenditures?
9. If so what are they?
10. How much are these other expenditures?

**Hospitalization Associated Expenditures**

Instructions to the patient: This is a series of questions related to how much money your household spent during this hospitalization for this patient. If you did not spend anything on the item in question, please state zero.

1. How much money did your household spend to transport the patient to the hospital?
2. How long did it take for the patient to reach the hospital once you started traveling?
3. How much money in total did your household spend for (all)/the attendant(s) to come to the hospital?
4. How much money did your household spend on medications?
5. How much money did your household spend on bandages and dressing supplies?
6. How much money did your household spend on laboratory tests?
7. How much money did your household spend on imaging and x-rays?
8. How much money did your household spend on any other medical supplies?
9. Did you have to pay any fees or make any informal payments directly to healthcare workers or hospital employees for your surgery or hospitalization? If so, how much did you pay? *Once again we want to emphasize that we are not recording your name, we do not need to know the name of anyone you paid money too, this is not for identification or blame. We are simply trying to understand how much patients have to pay for ALL types of costs.*
10. Did your household pay anyone else to act as an attendant and take care of the patient during this hospitalization?
11. Did your household have to borrow money to pay for this hospitalization?
12. Did your household have to sell and land or possessions (including livestock) to pay for this hospitalization?
13. Did your household have to stop sending any children to school, or did you pay reduced school fees in order to pay for this hospitalization?
14. How much did your household have to spend on food during this hospitalization?
15. How much in wages do you feel your household has lost due to this hospitalization?
16. Did you or anyone in your household lose your job as a result of this hospitalization?
17. How much did the social worker have to spend from the hospital fund to pay for your hospitalization for all goods and services?
